# Escore CHA2DS2-VASc: O Que Mais Podemos Predizer?

**DOI:** 10.36660/abc.20220672

**Published:** 2023-03-27

**Authors:** Adnaldo da Silveira Maia

**Affiliations:** 1 Instituto Dante Pazzanese de Cardiologia São Paulo SP Brasil Instituto Dante Pazzanese de Cardiologia, IDPC, São Paulo, SP – Brasil

**Keywords:** Fibrilação Atrial, COVID-19/complicações, Revascularização Miocárdica/métodos, Infarto do Miocárdio com Supradesnivelamento do Segmento ST, Mortalidade, Interpretação Estatística de Dados

**Caro editor**,

O trabalho realizado por Kalkan et al.,^1^ teve por objetivo avaliar o valor preditivo do escore CHA2DS2-VASc para o escore syntax residual (rSS) em pacientes com infarto agudo do miocárdio com supradesnivelamento do segmento ST (IAMCSST). 688 pacientes foram incluídos no estudo, os quais foram divididos em dois grupos a considerar o escore rSS: pontuação até 8: grupo de rSS baixo (grupo 1); pontuação acima de 8: grupo de rSS elevado (grupo 2).

Na análise geral dos pacientes, houve diferença estatística com relação a idade, dislipidemia, níveis de hemoglobina e glicose, lesão coronariana culpada e o escore CHA2DS2-VASc (p<0,001). A análise individual dos componentes do escore em cada grupo, revelou diferença nos seguintes: hipertensão arterial sistêmica, idade ≥ 75 anos, diabetes mellitus e doença vascular. Ademais, regressão logística realizada pelos autores identificou artéria coronária direita e escore CHA2DS2-VASc como fatores independentes de rSS elevado.

Desta forma, o estudo contribui para a discussão acerca da relevância do escore para predição do prognóstico de pacientes com rSS elevado no contexto de IAMCSST. No entanto, além desta importante casuística apresentada, outros trabalhos avaliaram a aplicabilidade do escore em contextos diferentes.

Sen et al.,^2^ avaliaram o valor preditivo do escore CHADS2 e CHA2DS2-VASc em pacientes com doença arterial coronária crônica em subanálise do COMPASS trial. Foram incluídos no estudo, 18.278 pacientes, tendo como desfecho primário desta análise, a ocorrência de eventos cardiovasculares maiores. Para ambos os escores avaliados, a ocorrência do desfecho primário foi superior à proporção da elevação dos mesmos. Além disso, a mortalidade por todas as causas, por causa cardiovascular, infarto e acidente vascular cerebral apresentaram diferença estatisticamente significativa para ambos os escores.

De forma semelhante, Burgos et al.,^3^ em estudo retrospectivo compararam a performance dos escores CHA2DS2-VASc, HATCH e POAF na predição de fibrilação atrial no pós-operatório de cirurgia cardiovascular. 3113 pacientes foram incluídos, entre os quais, cirurgia de revascularização miocárdica (45%), cirurgia valvar (24%), cirurgia combinada (15%) e outros procedimentos (16%) perpassaram a análise. Todos os escores revelaram boa performance na predição de fibrilação atrial no contexto de pós-operatório, sendo, segundo os autores, o escore CHA2DS2-VASc foi aquele com melhor poder discriminativo para fibrilação atrial.

Ainda neste âmbito, Tasbulak et al.,^4^ avaliaram em estudo retrospectivo de caso-controle com 280 pacientes a aplicabilidade dos escore CHA2DS2-VASc na detecção de oclusão dos enxertos utilizados na cirurgia de revascularização miocárdica. Considerou-se como falha dos enxertos, a presença de 70% ou mais de estenose bem como oclusão evidenciada à cinecoronarioangiografia. Na descrição geral dos pacientes, observou-se diferença estatística àqueles com hipertensão arterial sistêmica (p=0,004), diabetes mellitus (p=0,0001), níveis de creatinina (p=0,0001) e nos valores dos escores CHADS2 e CHA2DS2-VASc (0,0001). Regressão logística foi realizada para identificar fatores associados à falha dos enxertos, onde o escore CHA2DS2-VASc foi identificado como fator independente (OR 2,28) (95% IC 1,02-5,09).

Nos últimos 2 anos, a emergência da pandemia por COVID-19 e suas consequências ao sistema de saúde nacional trouxeram inúmeros desafios à tona. No entanto, também se ampliou o conhecimento acerca desta doença. Em publicação recente, Montazeri et al.,^5^ em estudo retrospectivo avaliaram o uso dos escores CHADS2 e CHA2DS2-VASc na predição de desfechos em pacientes acometidos por COVID-19.

Foram incluídos 1.406 pacientes com idade média de 59,47 ± 16,48, onde 60,46% eram do sexo masculino. Como desfecho primário, considerou-se mortalidade por todas as causas em três meses. Após análise dos dados, os escores foram capazes de predizer mortalidade, síndrome respiratória aguda, disfunção renal aguda e necessidade de ventilação mecânica.^5^

Em suma, a aplicabilidade abrangente, especialmente do escore CHA2DS2-VASc é notória, em diferentes contextos pode-se observar relação direta com determinados desfechos assim como prognóstico dos pacientes.

## References

[1] Kalkan AK, Kahraman S, Avci Y, Bulut U, Gulmea R, Turkyilmaz SB (2022). The Predictive Value of CHA2DS2-VASc Score on Residual Syntax Score in Patients With ST Segment Elevation Myocardial Infarction. Arq Bras Cardiol.

[2] Sen J, Tonkin A, Varigs J, Fonguh S, Berkowitz SD, Yusuf S (2021). Risk stratification of cardiovascular complications using CHA2DS2-VASc and CHADS2 scores in chronic atherosclerotic cardiovascular disease. Int J Cardiol.

[3] Burgos LM, Seoane L, Parodi JB, Espinoza J, Galizia Brito V, Benzadón M, Navia D (2019). Postoperative atrial fibrillation is associated with higher scores on predictive indices. J Thorac Cardiovasc Surg.

[4] Tasbulak O, Sahin A (2022). The CHA2DS2-VASc Score as an Early Predictor of Graft Failure After Coronary Artery Bypass Surgery. Cureus.

[5] Montazeri M, Keykhaei M, Rashedi S, Karbalai Saleh S, Pazoki M (2022). Prognostic significance of CHADS2 and CHA2DS2-VASc scores to predict unfavorable outcomes in hospitalized patients with COVID-19. J Cardiovasc Thorac Res.

