# Supplementary material for: Escore CHA2DS2-VASc: O Que Mais Podemos Predizer?
Source: Arq Bras Cardiol. 2023 Mar 27;120(4):e20220672. [Article in Portuguese] doi: 10.36660/abc.20220672 (PMC10263420; doi:10.36660/abc.20220672)
Supplement: Translation

# Translation: CHA_2_DS_2_-VASc Score: What More Can it Predict?

**Authors:** Adnaldo da Silveira Maia

**Affiliations:** 1 São Paulo SP Brazil Instituto Dante Pazzanese de Cardiologia, IDPC, São Paulo, SP – Brazil

**Keywords:** Atrial Fibrillation, COVID-19/complications, Myocardial Revascularization, ST Elevation Myocardial Infarction, Data Interpretation, Statistical

**Dear Editor,**

Kalkan et al.^1^ assessed the predictive value of the congestive heart failure, hypertension, age, diabetes mellitus, prior stroke, vascular disease, age, sex category (CHA_2_DS_2_-VASc) score for residual SYNTAX score (rSS) among patients with ST-segment elevation acute myocardial infarction (STEMI). The study included 688 patients divided into a low rSS group (group 1; scores up to 8) and a high rSS group (group 2; scores above 8).

The overall patient analysis showed statistically significant differences in age, dyslipidemia, hemoglobin and glucose levels, culprit coronary lesions, and CHA_2_DS_2_-VASc score (p<0.001). The individual analysis of the score components in each group revealed differences in systemic arterial hypertension, age ≥75 years, diabetes mellitus, and vascular disease. Furthermore, a logistic regression analysis identified the right coronary artery and CHA_2_DS_2_-VASc score as independent factors for a high rSS.

Therefore, the study contributes to the discussion on the relevance of the CHA_2_DS_2_-VASc score for predicting the prognosis of patients with a high rSS in the STEMI context. In addition to this important case series, other studies have assessed the score’s predictive value in other contexts.

Sen et al.^2^ assessed the predictive value of the CHADS_2_ and CHA_2_DS_2_-VASc scores in patients with chronic coronary artery disease in a subanalysis of the COMPASS clinical trial. The study included 18,278 patients, and its primary endpoint was the occurrence of major adverse cardiovascular events. For both scores, the primary endpoint occurred more frequently than a score increase. In addition, all-cause mortality, cardiovascular mortality, infarction, and strokes differed significantly for both scores.

Similarly, Burgos et al.^3^ retrospectively compared the performance of the CHA_2_DS_2_-VASc, HATCH (Hypertension, Age, Transient ischemic attack or stroke, Chronic obstructive pulmonary disease, and Heart failure), and POAF (PostOperative Atrial Fibrillation) scores for predicting atrial fibrillation after cardiovascular surgery. The study included 3,113 patients who underwent coronary artery bypass graft surgery (45%), valve surgery (24%), combined surgery (15%), and other procedures (16%). All scores showed a good ability to predict postoperative atrial fibrillation; the CHA_2_DS_2_-VASc score had the best discriminative power.

In the same context, Tasbulak et al.^4^ performed a retrospective case-control study of 280 patients to assess the predictive value of the CHA_2_DS_2_-VASc score for detecting coronary artery bypass graft occlusion. Graft failure was identified in cases of 70% or more graft stenosis and occlusion observed on coronary angiography. The general patient characteristics differed significantly for those with systemic arterial hypertension (p=0.004), diabetes mellitus (p=0.0001), creatinine levels (p=0.0001), and CHADS_2_ and CHA_2_DS_2_-VASc scores (p=0.0001). A logistic regression analysis identified that the CHA_2_DS_2_-VASc score was an independent factor for graft failure (odds ratio, 2.28; 95% CI, 1.02–5.09).

The emergence of the coronavirus disease 2019 global pandemic and its consequences for the national health system have created many challenges in the past 2 years. However, knowledge of this disease has also increased. Montazeri et al.^5^ retrospectively assessed using the CHADS_2_ and CHA_2_DS_2_-VASc scores for predicting outcomes in patients with coronavirus disease 2019.

The study included 1,406 patients with a mean age of 59.47 ± 16.48 years, 60.46% male. The primary outcome was 3-month all-cause mortality. The data analysis revealed that the scores could predict mortality, acute respiratory syndrome, acute renal dysfunction, and the need for mechanical ventilation.^5^

In conclusion, the broad predictive value of the CHA_2_DS_2_-VASc score is evident, and a direct association with certain outcomes and patient prognosis is observable in different contexts.
